# Identification and characterization of the *Plasmodium vivax *thrombospondin-related apical merozoite protein

**DOI:** 10.1186/1475-2875-9-283

**Published:** 2010-10-13

**Authors:** Alvaro Mongui, Diana I Angel, Darwin A Moreno-Perez, Silvana Villarreal-Gonzalez, Hannia Almonacid, Magnolia Vanegas, Manuel A Patarroyo

**Affiliations:** 1 Fundación Instituto de Inmunología de Colombia (FIDIC), Carrera 50 No. 26-20, Bogotá, Colombia; 2 Universidad del Rosario, Calle 63D No. 24-31, Bogotá, Colombia

## Abstract

**Background:**

Malaria caused by *Plasmodium vivax *is a major public health problem worldwide that affects 70-80 million people in the Middle East, Asia, Western Pacific, South America and the Caribbean. Despite its epidemiological importance, few antigens from this parasite species have been characterized to date compared to *Plasmodium falciparum*, due in part to the difficulties of maintaining an *in vitro *culture of *P. vivax*. This study describes the identification of the *P. falciparum *thrombospondin-related apical merozoite protein homologue in *P. vivax *(PvTRAMP) and examines its potential to be further evaluated as vaccine candidate.

**Methods:**

The gene encoding PvTRAMP was identified through an extensive search of the databases hosting the genome sequence of *P. vivax*. Genes adjacent to *pvtramp *were identified *in silico *to determine the degree of similarity between the protein sequences encoded by equivalent chromosomic fragments in *P. falciparum *and *Plasmodium knowlesi*. The *pvtramp *gene was amplified from cDNA of *P. vivax *schizont stages, cloned and expressed in *Escherichia coli*. Anti-PvTRAMP antisera was obtained by inoculating rabbits with PvTRAMP B cell epitopes produced as synthetic peptides in order to assess its recognition in parasite lysates by Western blot and in intact parasites by indirect immunofluorescence. The recognition of recombinant PvTRAMP by sera from *P. vivax-*infected individuals living in endemic areas was also assessed by ELISA.

**Results:**

The PfTRAMP homologue in *P. vivax*, here denoted as PvTRAMP, is a 340-amino-acid long antigen encoded by a single exon that could have a potential role in cytoadherence, as indicated by the presence of a thrombospondin structural homology repeat (TSR) domain. According to its transcription and expression profile, PvTRAMP is initially located at the parasite's apical end and later on the parasite surface. Recombinant PvTRAMP is recognized by sera from infected patients, therefore, indicating that it is targeted by the immune system during a natural infection with *P. vivax.*

**Conclusions:**

The results of this work support conducting further studies with PvTRAMP to evaluate its immunogenicity and protection-inducing ability in the *Aotus *animal model.

## Background

Five *Plasmodium *species are known to cause malaria in humans: *Plasmodium falciparum*, *Plasmodium vivax*, *Plasmodium malariae*, *Plasmodium ovale *and *Plasmodium knowlesi*. Of these five species, *P. falciparum *and *P. vivax *have the largest epidemiological impact, with *P. falciparum *malaria accounting for over 1 million deaths and around 300 million new cases annually [1], while *P. vivax *malaria has long been neglected and mistakenly considered a "benign" disease, but it is now gaining importance as it causes a considerable number of cases (~70-80 million cases per year), especially in the Middle East, Asia, the Western Pacific, South America and the Caribbean. *Plasmodium vivax *statistics could be even larger because it easily be misdiagnosed in endemic areas, where co-infection is frequent [2,3].

Knowledge on *P. vivax *has been considerably delayed compared to *P. falciparum *due to its tropism for reticulocytes, which account for less than 1% of the total circulating red blood cell (RBC) count and, therefore, makes it difficult to maintain an *in vitro *culture of this species [4]. However, the release of the complete genome sequences of *P. falciparum *and *P. vivax *[5,6], their corresponding transcriptome profiles [7,8], and the analysis of the *P. falciparum *proteome [9,10] have promoted studies aimed at identifying proteins involved in parasite invasion to host cells, such as proteins of the secretory apical organelles and surface proteins of blood-stage parasites. Based on this large body of available data, recent research in *P. vivax *malaria has made use of bioinformatics tools to identify and characterize new potential anti-malarial vaccine candidates by homology comparison (e.g. *P. vivax *vs. *P. falciparum*) [11-14].

The apical organelle complex of merozoites (the parasite's blood stage responsible for RBC invasion) plays an important role in parasite entry into RBCs. Tubular secretory structures, known as micronemes, belong to this complex; these secrete large amounts of proteins that are used by the parasite to adhere and invade host cells selectively [15-18]. It has recently been found that several of these invasion-related proteins contain domains that are highly conserved among the different apicomplexan species, such as the thrombospondin structural homology repeat (TSR) domain. TSR domains usually bind to highly variable sulfated glycoconjugates on the host cell surface that mediate cell-cell and/or cell-matrix interactions, therefore, conferring better host-cell binding specificity [19-22]. In essence, TSR domains are crucial for many biological processes and their functional relevance has been suggested based on the identification of this domain in relatively distant organisms [22].

To date, several apicomplexan antigens have been associated to the TSR family [20]. In *Plasmodium*, the most well characterized family members are found in sporozoites and include: the thrombospondin-related adhesive protein (TRAP), which is a conserved microneme protein important for parasite motility and for the infection of mosquito salivary glands and human hepatocytes [23]; the circumsporozoite protein (CSP), which is the most important sporozoite surface protein and is involved in gliding motility, among other functions; the CS related-TRAP protein (CTRP), which is an ookinete protein important for invasion of mosquito midguts [20,24]; PfSPATR, which contains an altered thrombospondin Type I repeat domain important for hepatocyte invasion and is expressed both on sporozoite surface and the micronemes of merozoites [25]; and finally, PTRAMP (initially identified in *P. falciparum *and here denoted as PfTRAMP), which has been shown to be expressed during the asexual intraerythrocytic cycle, with a maximum transcription peak during late schizonts. Besides containing a conventional TSR domain, PfTRAMP has an apical localization pattern in early schizonts and is later relocated to the surface of merozoites prior to schizont rupture.

Based on the importance of *Plasmodium *thrombospondin-like proteins and considering the results regarding the expression, transcription profile and cellular localization of PfTRAMP, as well as the *in vitro *affinity of chemically-synthesized peptides of PfTRAMP to RBCs [26,27], the aim of this study was to identify and characterize the PfTRAMP homologue in *P. vivax*: PvTRAMP, and conduct studies to determine its antigenicity.

## Methods

### Source of *P. vivax *parasites

The Vivax Colombia Guaviare 1 (VCG-1) strain was used as a source of *P. vivax *DNA, RNA, and proteins. In brief, parasites were cultured *in vivo *by successive passes in splenectomized *Aotus *monkeys [28] captured in the Amazon jungle with permission of the Colombian government (CORPOAMAZONIA) and kept at FIDIC's primate station in Leticia (Amazonas, Colombia). Monkeys were taken care of according to the guidelines stipulated by the Colombian Ministry of Health (law 84/1989) and the Office for Protection from Research Risks (OPRR, Department of Health and Human Service, USA), under the constant supervision of a primatologist. Levels of parasitaemia were assessed daily by Acridine Orange staining and monkeys were immediately treated whenever levels were ≥5%, or before if the monkey's health condition had deteriorated. Treatment consisted of orally administered paediatric doses of anti-malarial drugs as follows: 10 mg/kg of chloroquine on the first day and 7.5 mg/kg per day until day five, administered with 0.25 mg/kg of primaquine starting on day 3 and continuing until day 5. Once total clearance of parasites from blood had been confirmed and monkeys were in a satisfactory health conditions, they were released back into their natural habitat close to the site where they had been captured, with the supervision of CORPOAMAZONIA officials.

### Isolation of parasite genomic DNA and *in silico *gene analysis

Late-stage infected RBCs (mainly schizonts) were isolated from 3-4 mL samples of monkey blood using a discontinuous Percoll gradient, as previously reported elsewhere [29]. Genomic DNA was extracted from *P. vivax *infected-RBCs using the Wizard DNA purification system kit (Promega, Wisconsin, USA) and used as template for PCR amplification. Primers were designed based on the sequence of a putative transcript encoding the PfTRAMP homologue in *P. vivax*, which was identified by screening the genome sequence of the *P. vivax *Sal-1 strain, available at the JCVI website [30], using the amino acid sequence of the PfTRAMP as BLAST query. The databases hosting the complete genome sequences of *P. falciparum *and *P. knowlesi *were also screened with GenScan and GeneComber [31,32] in order to identify open reading frames (ORFs) adjacent to *pftramp *and *pktramp*. Identity and similarity values of peptide sequences between *P. vivax*-*P. falciparum *and *P. vivax*-*P. knowlesi *were obtained using the ALignX tool from the VectorNTI Suite 9 bioinformatics software package (Invitrogen, California, USA).

### RNA extraction, cDNA synthesis, cloning and sequencing

Total parasite RNA (1 μg) was extracted by the Trizol method [33] and subsequently treated with RQ1 RNase free DNase (Promega, Wisconsin, USA). A 20 μL aliquot of RNA was used a template for RT-PCR cDNA synthesis using the SuperScript III enzyme (Invitrogen, California, USA), according to manufacturer's recommendations. Briefly, cDNA was synthesized for 60 min at 50°C and then amplified by PCR with Platinum *Pfx *DNA polymerase (Invitrogen, California, USA) using *pvtramp *specific forward (5'-atgTACATTTTGCAGTTGCTCC-3') and reverse (5'-TTCATACATAAATCTGCCAGC-3') primers, for 35 cycles at the following temperatures: 94°C for 15 s, 54°C for 30 s, 68°C for 60 s and a final 5 min extension at 68°C. Primers were designed to exclude the first 29 amino acids of PvTRAMP. An additional PCR was carried out using non-reverse transcribed RNA as template to be used as negative control and rule out genomic DNA contamination. RT-PCR products were cloned into the pEXP5-CT/TOPO vector (Invitrogen, California, USA). Recombinant plasmid DNA was purified using the UltraClean mini plasmid prep purification kit (MO BIO laboratories, California, USA) and the integrity of the cloned insert was confirmed by sequencing of two clones obtained from independent PCR assays using an automatic ABI PRISM 310 Genetic Analyzer (PE Applied Biosystems, California, USA). The obtained nucleotide sequences, as well as their corresponding amino acid sequences, were aligned to the hypothetical *pvtramp *sequence reported for the Sal-1 reference strain.

### Expression and purification of rPvTRAMP

Recombinant PvTRAMP (rPvTRAMP) was obtained by cloning the *pvtramp *gene into the pEXP5-CT/TOPO vector. A six-histidine tag was added to the protein's C-terminus to allow purification and immunodetection by anti-histidine monoclonal antibodies. Briefly, *Escherichia coli *BL21-AI (Invitrogen) transformed with the *pvtramp*-pEXP5 vector were grown in 1 L of Terrific Broth containing 0.1 mg/mL ampicillin. Expression of PvTRAMP was induced by adding 0.2% arabinose (*w*/*v*) and incubating cells for 5 h at 37°C. Bacteria were pelleted by centrifugation at 12,000 × *g *for 30 min at 4°C and then resuspended in extraction buffer containing 6-8 M urea, 15 mM imidazole, 10 mM Tris-Cl, 100 mM NaH_2_PO_4_, 10 mg/mL lysozyme and protease inhibitors and lysed by sonication. To isolate rPvTRAMP, the lysate was centrifuged at 12,000 × *g *for 30 min at 4°C and the supernatant was mixed with Ni^+2^-NTA resin (Qiagen, California, USA), previously balanced to pH 8.0 with extraction buffer. Non-retained proteins were eluted with the same buffer, while the rPvTRAMP_6his _was eluted from the resin using extraction buffer with increasing concentrations of imidazole (50 mM, 100 mM, 200 mM and 500 mM). All fractions were analysed by sodium dodecyl sulfate polyacrylamide gel electrophoresis (SDS-PAGE) and Western blotting (see below). Pure protein fractions were pooled and dialyzed against 1 × PBS, pH 5.5, as a protein refolding step. The total amount of protein was determined by the bicinchoninic acid assay (BCA).

### Peptide synthesis

B cell linear epitopes were searched for in the putative sequence of *P. vivax *Sal-1 PvTRAMP using BepiPred at a default threshold of 0.35 and 75% of specificity [34]. Taking into account Parker's antigenicity, solvent accessibility and hydrophobicity values from ANTHEPROT [35], three 20-mer-long peptides were selected for peptide synthesis. The sequences of these peptides in single letter code were: ^51^AVGAGSQLGQAAQESDVNRK^70^, ^82^NSQFTNEKVLEVYSSKEENV^101 ^and ^155^DVKNPSEFEILSEPIKFSIS^174^. Peptides were synthesized by standard solid-phase *t*-Boc/Bzl peptide synthesis [36], adding glycine and cysteine residues at the N- and C-terminal ends, respectively, to allow polymerization. The peptides were lyophilized and then characterized by RP-HPLC and MALDI-TOF MS.

### Polyclonal antibodies against rPvTRAMP

Three New Zealand rabbits were subcutaneously inoculated at multiple sites with the three PvTRAMP B cell linear epitopes mentioned above. Peptides were administered as a 150 μg mixture of polymerized synthetic peptides, emulsified in Freund's complete adjuvant (FCA) for the first dose (day 0) and in Freund's incomplete adjuvant (FIA) for the second and third doses (days 21 and 42). Sera were collected before the first inoculation to obtain pre-immune sera and 21 days after the third immunization to obtain hyper-immune sera.

### SDS-PAGE and Western blotting

Malarial parasites isolated from total blood samples of *P. vivax*-infected *Aotus *monkeys were lysed with 20% SDS, 0.5 M EDTA, 100 mM PMSF, and 100 mM iodoacetamide. Proteins in the parasite lysate were separated by 12% SDS-PAGE and electro-transferred to nitrocellulose membrane. Purified recombinant protein was loaded into the same gel as positive control. The membrane was blocked with 5% skimmed milk in PBS-0.05% Tween, washed thrice with PBS-0.05% Tween for 5 min and then cut into strips to assess recognition by each rabbit's serum. Strips were incubated with a 1:40 dilution of rabbit sera for 1 h at room temperature, then washed thrice with PBS-0.05% Tween and incubated at room temperature for 1 h with 1:4,500 alkaline phosphatase-coupled goat anti-rabbit IgG. Excess of antibody was removed by washing strips thrice with PBS-0.05% Tween and color development was assessed using the BCIP/NBT kit (Promega, Madison, USA), according to manufacturer's instructions. The positive control strip was incubated for 1 h with 1:4,500 peroxidase coupled anti-polyhistidine monoclonal antibody diluted in 5% skimmed milk-PBS-0.05% Tween, washed thrice with PBS-0.05% Tween and then treated with VIP peroxidase substrate (Vector Laboratories, Burlingame, Canada).

### Recognition of rPvTRAMP by sera from *P. vivax*-infected individuals

The recognition of rPvTRAMP by sera from *P. vivax *infected individuals was assessed by ELISA. In brief, sera were collected from 20 individuals who inhabited a *P. vivax *malaria endemic area in Colombia and had an active episode of *P. vivax *malaria at the time of blood withdrawal, as well as from three healthy individuals who lived in a non-endemic area and had never had an episode of *P. vivax *malaria (negative controls). All individuals were explained about the objective of the study and signed an informed consent form before blood withdrawal. All procedures were evaluated and approved by FIDIC's ethics committee. Polysorb plates were coated with purified rPvTRAMP (1 μg/well), incubated first at 37°C for 1 h, then at 4°C overnight and once more at 37°C for 1 h. Each well was washed thrice with PBS-0.05% Tween and then blocked with 200 μL of 5% skimmed milk in PBS-0.05% Tween for 1 h. Sera were added to the plates in duplicate as a 1:100 dilution in 5% skimmed milk PBS-0.05% Tween and incubated at 37°C for 1 h. Plates were then washed thrice with PBS-0.05% Tween and incubated for 1 h at 37°C with 100 μL of a 1:4,000 dilution of peroxidase conjugated anti-human IgG as secondary antibody. Excess of peroxidase-coupled antibody was removed by washing plates thrice with PBS-0.05% Tween. Colour development was evaluated using the TMB Microwell Peroxidase Substrate solution kit KPL Laboratories, Washington, USA) and optical density (OD) measures at 620 nm using a microplate reader (Labsystems Multiskan MJ ELISA reader).

### Indirect immunofluorescence assays

Thin blood smears of *P. vivax*-infected monkeys (3-5% parasitaemia) were prepared on glass slides, then fixed with acetone/methanol, chilled at 4°C and blocked PBS-10% Fetal Calf Serum (FCS) for 1 h at 37°C. Slides were then incubated inside a humid chamber for 1 h at 37°C with primary antibody (rabbit anti-synthetic peptides' polyclonal antibodies) diluted to 1:20 in blocking buffer, then washed thrice with PBS and incubated with a 1:40 dilution of goat anti-rabbit IgG-fluorescein isothiocyanate conjugate (Sigma, Missouri, USA). Finally, slides were stained with 2 μg/mL of DAPI for 20 min at room temperature and examined using an Olympus BX51 fluorescence microscope.

## Results and Discussion

### Identification and characterization of the gene encoding TRAMP in *P. vivax*

A PfTRAMP homologue was identified in *P. *vivax by screening the entire *P. vivax *genome sequence retrieved from the JCVI database using the PfTRAMP sequence [GenBank: AAN36262.1, PlasmoDB: PFL0870w] as query for tBLASTn analyses. The *pvtramp *gene is located in a 3,120,417 bp chromosomic segment [PlasmoDB: CM000455], which contains a 1,023-bp ORF [PlasmoDB: PVX_123575], encoding a 340 amino-acid-long protein with a theoretical mass of 39 kDa.

**Figure 1:**
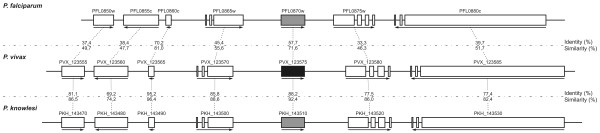
**Schematic representation of *pftramp*, *pvtramp *and *pktramp***. The figure shows the localization of the genes encoding PfTRAMP, PkTRAMP (both in gray) and PvTRAMP (in black) in *P. falciparum, P. knowlesi *and *P. vivax *chromosomic fragments, respectively, as well as the localization of the adjacent genes evaluated in this study. The arrows above each box indicate the ORF orientation, while the boxes show distribution and organization of the exons along the chromosomal segments. Genes are assigned according to their annotation in PlasmoDB. These chromosomic fragments comprised 21.5 kbp from the *P. falciparum *chromosome 12 (695,001-716,500 pb), 23.5 kbp from the *P. vivax *contig CM000455 (1,519,501-1,543,000 pb) and 24 kbp from the *P. knowlesi *chromosome 14 (1,548,001-1,572,000 pb).

Additionally, a search for genes orthologous to *pftramp *on the PlasmoDB protein database identified similar genes in *Plasmodium chabaudi *(*Pch*), *Plasmodium berghei *(*Pb*), *Plasmodium yoelii *(*Py*) and *Plasmodium knowlesi *(*Pk*). Based on a previous phylogenetic analyses showing shorter divergence time between *P. vivax *and *P. knowlesi *[37], this latter species was chosen, together with *P. falciparum*, to compare the chromosomic region containing the putative *pvtramp *gene. The alignment showed homology between the ORFs encoding PvTRAMP and PfTRAMP, as well as high overall identity (Id) and similarity (S) values between the protein sequences derived from *pvtramp*, *pftramp *and *pktramp *(Id = 54.55% and S = 83.25%). To confirm gene/chromosome synteny between the 3 species, the chromosomic regions containing the three *tramp *genes were compared (23.5 kbp in *P. vivax, *21.5 kbp in *P. falciparum *and 24 kbp in *P. knowlesi*), observing that the upstream and downstream orientation of ORFs as well as the exon-intron composition are conserved among the three species. This is consistent with identity and similarity values obtained when comparing the three *tramp *genes as well as the peptide sequences derived from genes adjacent to *pvtramp*, *pftramp *and *pktramp*, which showed Id and S values of 33.3-70.2% and 46.3-81%, respectively between *P. vivax*-*P. falciparum*, and Id and S values of 69.2-95.2% and 74.2-96.4% between *P. vivax*-*P. knowlesi*, respectively (Figure 1).

**Figure 2:**
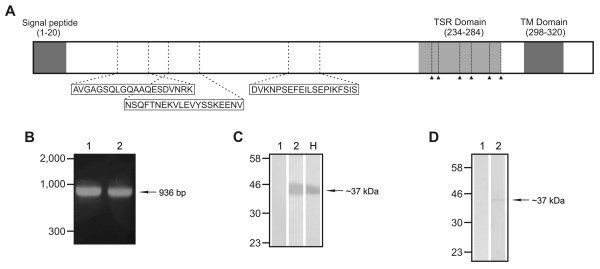
**(A) Schematic representation of PvTRAMP indicating the localizations of the predicted signal peptide and transmembrane domain (both in dark gray), as well as the TSR domain (light gray)**. Localization of the conserved cysteines inside the TSR domain and the synthetic peptides used in this study to obtain anti-PvTRAMP antisera are indicated by arrow heads and white boxes, respectively. **(B) **PCR amplification of *pvtramp *from *P. vivax *genomic DNA and cDNA. Lane 1. Amplification from genomic DNA using primers designed based on the sequence predicted for *pvtramp*. Lane 2. RT-PCR product amplified from DNAse-treated total *P. vivax *RNA. **(C) **Recognition of purified rPvTRAMP by anti-PvTRAMP rabbit sera, as assessed by Western blot. Lane 1: pre-immune sera. Lane 2: hyperimmune sera. Lane H: recognition of purified rPvTRAMP by anti-polyhistidine monoclonal antibody. **(D) **Western blot analysis of a *P. vivax *lysate with anti-PvTRAMP rabbit sera. Lane 1: pre-immune sera. Lane 2: hyperimmune sera.

PvTRAMP is 12 residues shorter than its homologue PfTRAMP (41 kDa) and contains a hydrophobic region at its N-terminus, which is consistent with a signal peptide according to SignalP v3.0 [38]. In PvTRAMP the signal peptide cleavage site lies between amino acids 20 and 21 (AIS-EK) (Figure 2a), while in PfTRAMP it lies between amino acids 24 and 25 (ISS-ND). PvTRAMP seems to have a C-terminal transmembrane domain (TM) according to the TMHMM v.2.0 [39] prediction server hosted at the Center for Biological Sequence Analysis of the Technical University of Denmark [40], which had been also predicted for PfTRAMP [26].

A single TSR domain was detected in PvTRAMP when the sequence was analyzed using the InterProScan [InterPro: IPR000884] and PFAM [PFAM: PF00090] databases. This TSR domain has a high significance value and spans from residue 234 to 284, thus lying close to the TM domain (Figure 2a). In general, it has approximately 50-60 amino acids in length, and contains 12 or more highly conserved and typically separated residues comprising 6 cysteines (C), 2 to 3 tryptophans (W) and 2 arginines (R) [19]. The crystal structure showed that this domain is divided into two sub-groups (1 and 2) depending on the rearrangements between conserved cysteines and the disposition of aromatic residues, which determine the folding of the major helix in the protein's structure [26]. The TSR domain is highly conserved among the different apicomplexan species and is characterized by the presence of one or multiple copies of type I human thrombospondin domain. In general, it has a distinctive amino acid sequence (EWSPCSVTCGXGXRXR), preceded by the sequence WX (where X is generally an acid residue), which is an interesting aspect since the presence of two conserved tryptophan residues is not very frequent in *Plasmodium *spp. [20,26]. It is important to mention that the main function of this domain lies in its amino-terminal motif which mediates binding to glycosaminoglycans (GAG); a characteristic that is associated with the invasion ability of parasite proteins containing TSR domains [23,41].

The disposition of the TSR domain of PvTRAMP is consistent with studies on PfTRAMP by Thompson *et al *[26], where it has been shown that the classical architecture of the domain is not altered by some residue substitutions. Same as other TSR domains, PvTRAMP has a characteristic arrangement of six cysteine residues defining three highly conserved motifs that are particularly significant for structure and binding functions and is conserved among *P. vivax*, *P. falciparum *and *P. knowlesi*. The first is an amino-terminal W**WG*W motif (where * represents any residue), although the first tryptophan is substituted by a tyrosine in PvTRAMP and PfTRAMP. The second motif, CS/T*TC, shows an unusual threonine → aspartic acid substitution, whose functional significance is still unclear. Finally, the third motif (I/R/Q*R*R) is thought to be intercalated between the conserved tryptophan residues and is also present in PvTRAMP [26].

### *pvtramp *is transcribed in parasite asexual stages

The RNA isolated from the parasite blood stages was treated with DNAse to avoid contamination with genomic DNA and used as template for reverse transcription. The sizes of the PCR and RT-PCR amplification products agreed with the size expected based on sequence for *pvtramp *without including the first 29 amino acids (963 bp) (Figure 2b). This result shows that PvTRAMP is encoded by a single 1,023-pb exon, which was confirmed by sequencing of genomic DNA and cDNA products from independents PCR assays.

No substitutions were found when comparing the sequences from the VCG-1 strain (*Aotus*-adapted strain) obtained by sequencing of the cloned products, compared to the sequence reported for the Sal-1 strain, suggesting, therefore, that *pvtramp *is highly conserved among isolates obtained from different geographic regions. However, this needs to be confirmed by comparing a larger number of strains [28].

### PvTRAMP is expressed in *P. vivax *asexual stages

Based on the PvTRAMP sequence reported for the Sal-1 strain, three 20 mer-long synthetic peptides were designed, taking care of selecting peptides outside the TSR domain (amino acids 232 to 285) in order to avoid cross-reactivity with other proteins containing TSR domains (Figure 2a). Immunochemistry assays with polyclonal sera obtained by immunizing three New Zealand rabbits with a mixture of these three peptides showed that PvTRAMP is expressed in late intraerythrocytic parasite stages (schizonts), as has been previously described for its PfTRAMP homologue [26]. Hyperimmune but not pre-immune polyclonal rabbit sera raised against the peptide mixture recognized a 37-kDa band by Western blot when purified recombinant PvTRAMP was size separated by 12% SDS-PAGE, which is consistent with the weight expected for PvTRAMP based on its sequence (Figure 2c). Likewise, the Western blot analysis of polyclonal rabbit sera against a schizont-rich *P. vivax *protein lysate showed recognition of a 37-kDa band by the hyperimmune sera but not by the pre-immune sera (Figure 2d).

**Figure 3:**
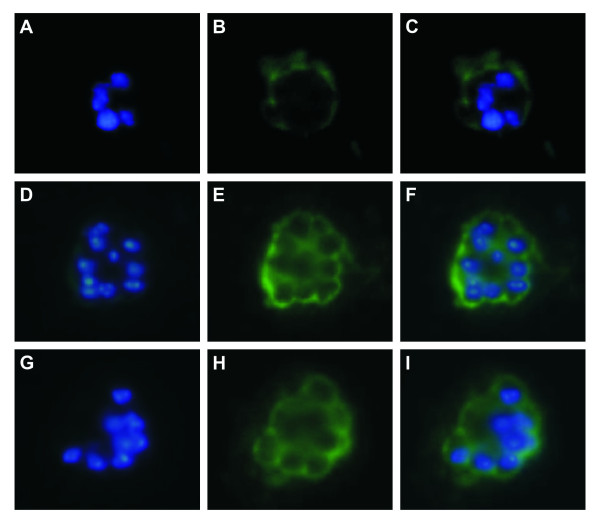
**Cellular localization of PvTRAMP as assessed by IFA using hyper-immune anti-PvTRAMP rabbit sera as primary antibody**. **(A-C) **Detection of *P. vivax *in early schizont stages. **(D-I) **Parasites in late schizont stage (segmented). The figure shows fluorescence with DAPI and FITC staining, and the merging of both.

Immunofluorescence assays using blood smears from *P. vivax-*infected *Aotus *monkeys and pooled rabbit hyperimmune sera as primary antibody detected PvTRAMP in schizont stages. As shown in Figure 3, a fluorescence pattern typical of merozoite surface proteins with some apparent concentration towards the apical pole was observed, which is similar to the fluorescence pattern reported for PfTRAMP. Localization assays performed by Thompson *et al *[26] using a combination of antisera against the rhoptry-associated protein RAP1 and the apical membrane antigen AMA1 showed that PfTRAMP co-localizes with AMA-1 in apical organelles from early schizonts, but displays a distinct localization pattern compared to RAP1 [26,42]. This has lead the authors to suggest that PfTRAMP originates at the micronemes and relocalizes to merozoite surface before initiation of RBC invasion. Further confocal microscopy studies with more specific antibodies would help defining whether *P. vivax *TRAMP exhibits this localization pattern.

### rPvTRAMP recognition by human sera

**Figure 4:**
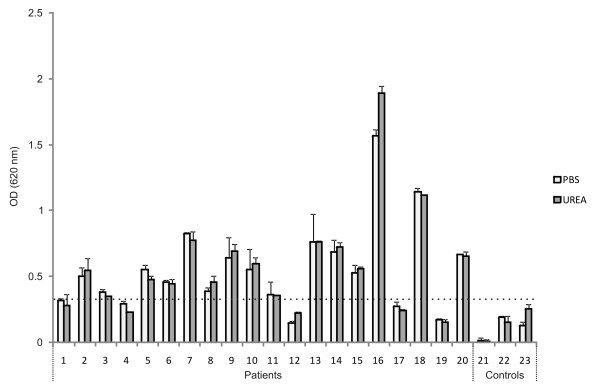
**ELISA showing reactivity of sera from *P. vivax*-infected patients against rPvTRAMP**. Columns 1-20 correspond to recognition by sera from *P. vivax *malaria patients. Columns 21-23 showed recognition of rPvTRAMP by healthy individuals that had never had an episode of *P. vivax *malaria. rPvTRAMP used in this assay was resuspended in urea and thoroughly dialyzed against PBS for its refolding. Each column is shown with its corresponding standard deviation.

An ELISA test was performed to evaluate the degree of recognition of rPvTRAMP by sera from 20 patients who had an active *P. vivax *infection at the moment of blood withdrawal and had had more than one episode of *P. vivax *malaria during their lifetime. Most sera showed a high recognition of rPvTRAMP with OD values that were significantly higher than the highest OD value obtained for the negative controls plus twice the standard deviation (Figure 4). However, for most sera, recognition of the refolded protein was not notably greater compared to recognition of the protein obtained under denaturing conditions. This may suggest that (1) PvTRAMP exhibits both linear and conformational epitopes during a natural infection, both of which have the potential to stimulate the host's immune response, or (2) that the refolding step was not sufficient for rPvTRAMP to fold back properly and therefore it did not acquire a 3D structure similar to that of the native protein expressed by the parasite (probably because of its high cysteine content), as the immune system's recognition of conformational epitopes is usually predominant for *Plasmodium *proteins involved in invasion [12,43,44]. Because of this, it would be important to obtain PvTRAMP in native conditions and performing functional assays to evaluate its activity.

## Conclusions

The transcription and expression profile of PvTRAMP, its merozoite surface localization and its high degree of conservation when compared with homologous proteins from other *Plasmodium *species have been determined in the present study. Moreover, the high yield obtained when expressing PvTRAMP as a recombinant protein and the broad recognition by sera from *P. vivax*-infected people support further studies aimed at evaluating its immunogenicity and protective ability (as a full protein or its derived synthetic peptides) in a relevant biological model, such as the *Aotus *monkey.

## Competing interests

The authors declare that they have no competing interests.

## Authors' contributions

AM designed experiments, analysed data and wrote the final manuscript. DIA carried out assays, interpreted the results and wrote the initial draft. DAMP performed IFA experiments, obtained all the polyclonal sera and tabulated the results. SVG carried out DNA extraction, PCR amplification and cloning assays. HA was in charge of expressing and purifying the recombinant protein. MV provided purified synthetic peptides for rabbit immunizations. MAP evaluated and coordinated assays, critically revised the manuscript and gave the final approval for its publication. All authors read and approved the final manuscript.
